# Probiotics modulate gastrointestinal microbiota after *Helicobacter pylori* eradication: A multicenter randomized double-blind placebo-controlled trial

**DOI:** 10.3389/fimmu.2022.1033063

**Published:** 2022-11-08

**Authors:** Cong He, Yong Xie, Yin Zhu, Kun Zhuang, Lijuan Huo, Yong Yu, Qiang Guo, Xu Shu, Zhijuan Xiong, Zhenyu Zhang, Bin Lyu, Nonghua Lu

**Affiliations:** ^1^ Department of Gastroenterology, Digestive Disease Hospital, The First Affiliated Hospital of Nanchang University, Jiangxi, China; ^2^ Jiangxi Clinical Research Center for Gastroenterology, Nanchang, China; ^3^ Department of Gastroenterology, Xi’an Central Hospital, Shaanxi, China; ^4^ Department of Gastroenterology, First Affiliated Hospital of Shanxi Medical University, Taiyuan, China; ^5^ Department of Gastroenterology, Fifth Affiliated Hospital of Zhengzhou University, Zhengzhou, China; ^6^ Department of Gastroenterology, First People’s Hospital of Yunnan Province, Kunming, China; ^7^ Department of Gastroenterology, Nanjing First Hospital, Nanjing Medical University, Jiangsu, China; ^8^ Department of Gastroenterology, First Affiliated Hospital of Zhejiang Chinese Medical University, Hangzhou, China

**Keywords:** helicobacter pylori eradication, probiotics, gut microbiota, gastric microbiota, saliva microbiota, 16S rRNA gene sequence

## Abstract

**Background:**

*Helicobacter pylori* (*H. pylori*) eradication has been reported to cause short-term disruption of gut microbiota. It is acknowledged that probiotics supplementation mitigates side effects induced by *H. pylori* eradication, yet its role on alleviating dysbiosis of microbiota is obscure.

**Objectives:**

To evaluate the impact of probiotics on gastrointestinal microbiota after eradication therapy.

**Methods:**

This was a multicenter, double-blinded, randomized trial done at seven centers in China. A total of 276 treatment-naïve *H. pylori*-positive patients were randomly assigned to receive 14-day bismuth-containing quadruple therapy (esomeprazole, bismuth, amoxicillin, furazolidone) combined with probiotics (Bifidobacterium Tetragenous viable Bacteria Tablets) (n=140) or placebo (n=136) for 28 days. Saliva, gastric mucosa and fecal samples were collected before and after therapy for 16S rRNA gene sequencing.

**Results:**

The incidence of gastrointestinal adverse events was lower in probiotics group compared to placebo group (23.6% vs 37.7%, p=0.016), while there was no significant difference in eradication rate. We found dramatic perturbations of gut microbiota immediately following eradication, with the predominance of Proteobacteria in replacement of commensal Firmicutes and Bacteroidetes, and gradually restored after two weeks. The reduction of gut Bacteroidetes caused by eradication drugs was neutralized with probiotics supplementation. The gastric microbiota was completely reconstituted with *H. pylori* depleted and other taxa flourished. Of note, patients treated with probiotics showed smaller fluctuations of gastric microbiota compared to those with placebo. We also observed changes of saliva microbiota after *H. pylori* eradication, illustrated by the overgrowth of *Neisseria* and depletion of *Streptococcus*. The expansion of some pathogenic genera, including *Porphyromonas*, *Leptotrichia*, in the mouth was suppressed by probiotics.

**Conclusion:**

This study not only demonstrated the beneficial effect of probiotics implementation on side events during *H. pylori* eradication but also provided a comprehensive profile of microbiome alterations along gastrointestinal tract that modulated by probiotics.

## Introduction

*Helicobacter pylori* (*H. pylori*), the pathogenic bacterium in the stomach, infects approximately 50% of the population worldwide and causes a wide range of gastric diseases, including chronic gastritis, peptic ulcer and gastric cancer. Thus, it is recommended to screen and eradicate *H. pylori* to prevent those dieseases (1, 2). With the decreased eradication rate of standard triple regimen nowadays, the first-line regimen for *H. pylori* treatment is bismuth-containing quadruple therapy, which includes bismuth, proton pump inhibitor, and two antibiotics, in a series of global consensus (1, 3, 4). Accumulating evidence suggest that eradication drugs, especially antibiotics, lead to the disorder of gut microbiota, whose homeostasis play an important role in human health (5). The composition of gut microbiota changed immediately after *H. pylori* treatment with reduced alpha diversity and increased abundance of *Enterobacteriaceae* and *Enterococcus* (6, 7). Although most reported full recovery of bacterial diversity six months after eradication, some studies observed notable changes in abundance at genus level (8). Therefore, it is critical to alleviate the profound impact of drugs involved in *H. pylori* therapy on gut microbiota.

Probiotics, as live micro-organisms, are commonly used to modulate gut microbiota and ameliorate human diseases like metabolic syndrome, colitis, and antibiotic-associated diarrhea (9). A large body of studies has shown that probiotics supplementation mitigates the side effects during *H. pylori* eradication, although with controversial effect on improving eradication rate (10, 11). Moreover, several randomized controlled trials have observed that probiotics supplementation help to construct a beneficial profile of gut microbiota after eradication therapy (12). While the alpha diversity was increased with the combination of non-viable *Lactobacillus reuteri*, no significant changes of diversity was found with Medilac-S supplementation (13). The inconsistency between those studies is probably due to different probiotic strains and load. In addition to gut microbiota, probiotics have been reported to restore the dysbiosis of gastric microbiota induced by *H. pylori* eradication (14). Thus, it is of interest to explore whether probiotics administration could influence microbiota along gastrointestinal tract in patients receiving eradication therapy.

The live probiotic drug Bifidobacterium Tetragenous viable Bacteria Tablets is comprised of four strains, including *Bifidobacterium infantis* CGMCC0460.1, *Lactobacillus acidophilus* CGMCC0460.2, *Enterococcus faecalis* CGMCC0460.3, and *Bacillus cereus* CGMCC0460.4. It has been widely applied to modulate intestinal dysbacteriosis for patients with diarrhea, constipation and dyspepsia. The numbers of probiotic bacteria *Bacteroides*, *Faecalibacterium* and *Akkermansia* were significantly enhanced after probiotic treatment, while the richness of pathogenic *Streptococcus* was lowered (15, 16). Herein, a randomized, double-blinded, placebo-controlled trial was conducted to evaluate the efficacy and safety of probiotics combined with 14-day bismuth-containing quadruple therapy on *H. pylori* eradication and the changes of gastrointestinal microbiota.

## Methods and materials

### Study design

This multicenter, randomized, placebo-controlled study was performed at seven tertiary hospitals in China from March 2019 to November 2021. This study was approved by the Ethics Committee of the First Affiliated Hospital of Nanchang University (2019-072) and registered at ClinicalTrials.gov (NCT04034641).

### Eligibility criteria

In brief, adult patients (18-65 years) diagnosed with chronic gastritis and tested positive for *H. pylori* were prospectively recruited. *H. pylori* infections were examined by the [^13^C] urea breath test. Patients with any one of the following criteria were excluded from the study: diagnosed with gastric cancer, peptic ulcer diseases, esophagitis, history of gastrectomy, previous eradication therapy for *H. pylori*, allergy to any of the medications, severe concurrent diseases, pregnant or lactating women, the use of antibiotics or probiotics in the preceding 4 weeks, alcohol abuse or drug addiction, and patients who could not give informed consent.

### Interventions

Eligible patients were randomly allocated to probiotics group (Group A) or the placebo group (Group B) in a 1:1 ratio. Randomization was conducted by a computer-generated random number sequence, and the assignments were sealed in opaque envelops. Patients, clinical researchers and statistician were blinded to the randomization and study products. Both groups of patients received 14-day bismuth-containing quadruple therapy (esomeprazole 20mg, amoxicillin 1000mg, furazolidone 100mg, bismuth potassium citrate 220mg, dosed morning and evening), supplemented with probiotics (Bifidobacterium Tetravaccine Tablets, SiLianKang, Hangzhou Grand Biologic Pharmaceutical Inc., Hangzhou, China) or placebo (starch) (nine tablets once a day, dosed noon) for 4 weeks.

### Procedures

**Figure 1:**
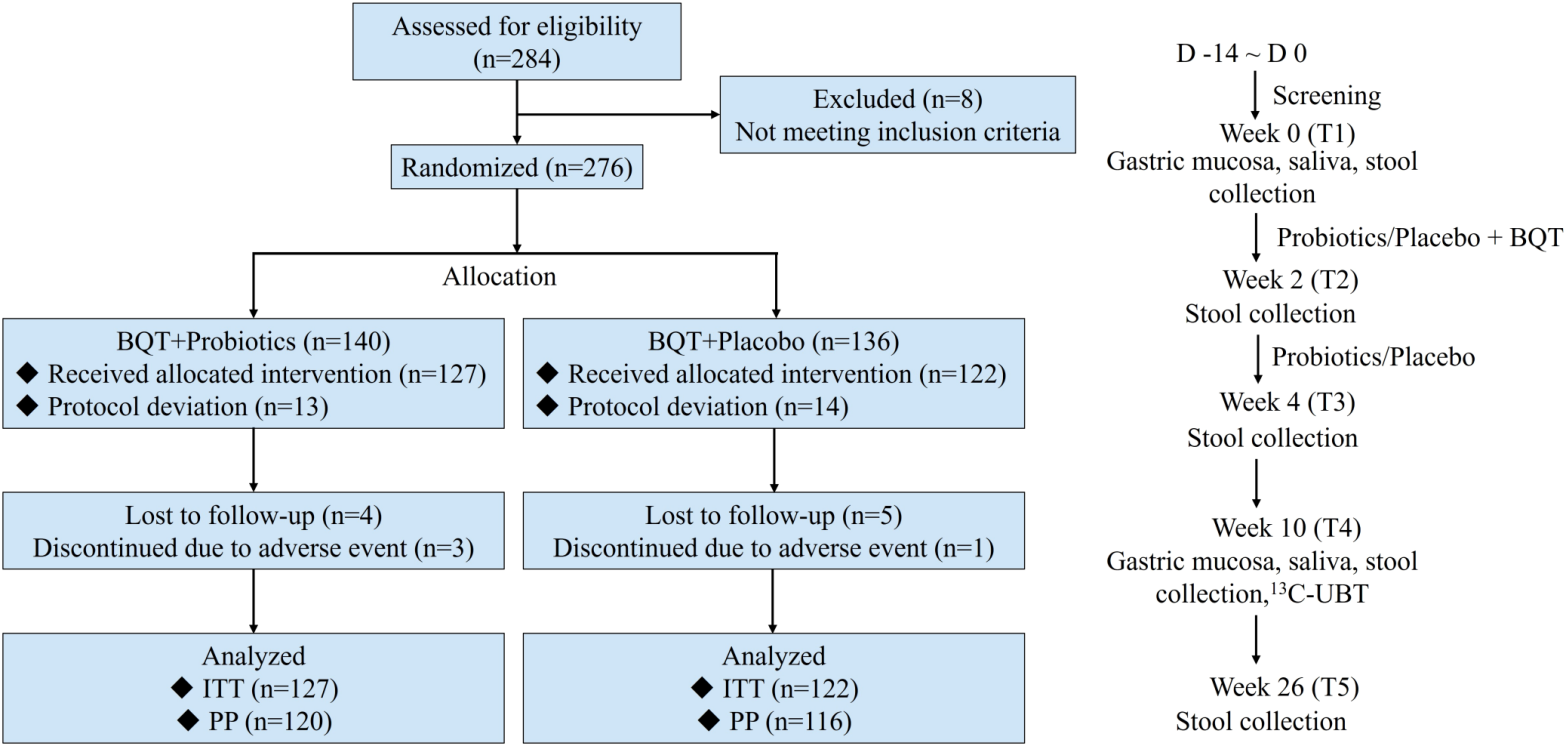
Flow diagram showing the study design. BQT, bismuth quadruple therapy; ITT, intention-to-treat; PP, per protocol; D, day; ^13^C-UBT, ^13^C-Urea Breath Test.

Patients were evaluated at 5 visits: screening at baseline (T1), end of 14-day eradication therapy (T2), end of probiotics/placebo treatment (T3), 8 weeks after eradication (T4), and 24 weeks after eradication (T5). Fecal samples were collected into a DNA stabilizer kit (Fecal DNA Storage Buffer, CoWin Biosciences Co. Ltd, China) from T1 to T5. The liquid DNA stabilization buffers protect the microorganisms from degradation. The stabilized samples were then stored in a refrigerator at -80°C immediately. The gastric antrum mucosa and saliva samples were collected at T1 and T4 and immediately frozen at -80°C. A flow diagram of this trial was shown in **Figure 1**. The OMEGA Soil DNA Kit (M5635-02) (Omega Bio-Tek, USA) was used to extract the genomic DNA. High throughput sequencing of 16S rRNA was done by means of the Illumina NovaSeq platform (Shanghai Personal Biotechnology Co., Ltd, China). Detailed methods are shown in the **Supplement Materials**.

### Outcomes

The primary outcome of this study was the incidence of gastrointestinal adverse events, including vomiting, bloating, diarrhea, etc. The second outcomes included eradication rate of *H. pylori*, compliance with therapy, alterations of gastrointestinal microbiota.

### Statistical analyses

Based on a literature review of *H. pylori* eradication-induced adverse events, we expected a difference between quadruple therapy combined with probiotics and placebo on the incidence of adverse events of 14.4% vs 30.1% (17). The calculation yielded 119 for probiotics group and 119 for placebo group, with a power of 80% and a two-sided significance level of 0.05 with an assumed 10% dropout rate.

Statistical analyses were performed using the software SAS (version 9.4). The data are presented as means ± standard deviations (SDs) or as percentages. The eradication rate was determined by both intention-to-treat (ITT)- and per-protocol (PP)-based analyses. All enrolled patients were included in the ITT analysis, but the PP analysis excluded those discontinued or loss to follow-up. We used the χ^2^ test or Fisher’s exact test for analysis of categorical data, and Student’s t test or Wilcoxon rank sum test for analysis of continuous data. All *P*-values were 2-tailed and *P* < 0.05 was considered as significant.

Microbiome bioinformatics were performed with QIIME2 and R packages (v3.2.0) (18). Alpha diversity of microbiota was determined by calculating indices, including Shannon and Chao1. Significant differences of beta diversity were evaluated by PERMANOVAR and visualized by Principal coordinate analysis on the basis of Bray-Curtis distance. Wilcoxon signed rank-sum test was used to analyze the differential taxa for paired samples. Co-occurrence network analysis was performed by SparCC and visualized using R package. Genera with significantly changed abundance in T4 were combined into Microbial Dysbiosis Index (MDI) for using follow formula: MDI=log(total abundance of genera decreased after *H. pylori* eradication/total abundance of genera increased after *H. pylori* eradication) (19). The difference between the MDI of the timepoint of T4 and the baseline T1 were defined as delta-MDI, which was calculated using the difference value of MDI between paired samples. Detailed methods are shown in the **Supplement Materials**.

## Results

### Trial profile

A total of 276 patients fulfilling the inclusion criteria were assigned randomly to either Group A (probiotics) or Group B (placebo). Of these 276 patients, 27 patients were excluded as they refused to take study drugs, and the remaining 249 patients were enrolled for intention-to-treat (ITT), with 127 patients in Group A and 122 patients in Group B. During the study, thirteen patients were excluded from the per-protocol (PP) analysis: 7 in Group A (5.5%) and 6 in Group B (4.9%). Three patients discontinued treatment and ten patients lost to follow-up (**Figure 1**). Demographic and baseline characteristics of the patients in the two groups are shown in **Supplement Table 1**.

### *H. pylori* eradication rate and adverse events

As shown in **Table 1**, ITT analysis demonstrated that the eradication rates were 86.6% for Group A and 87.7% for Group B (p=0.797). PP analysis indicated that the eradication results were 91.7% for Group A and 92.2% for Group B (p=0.871). Both ITT and PP analyses showed no statistical difference in the eradication rate between Group A and Group B.

**Table 1:** Eradication rate of *H. pylori* in patients treated with probiotics (A) or placebo (B).

Group	Eradication rate, %(n) (95% confidence interval) ITT analysis	PP analysis
Group A	86.6 (11./127) (80.6-92.6)	91.7 (110/120) (86.6-96.7)
Group B	87.8 (107/122) (81.8-93.6)	92.2 (107/116) (87.3-97.2)
*P* value	0.797	0.871

The frequencies of gastrointestinal adverse events (GAE) were summarized in **Table 2**. All of the GAE were graded as mild. The overall incidences of GAEs were significantly reduced in Group A compared to Group B (23.62% vs 37.7%, p=0.016). The nausea rate of Group B was higher than that of Group A (24.59% vs 13.39%, p=0.024). Meanwhile, the combination of probiotics tended to decrease the incidence of vomiting (2.36% vs 7.38%, p=0.065). There was no statistical difference between Group A and Group B in terms of diarrhea, bloating, abdominal pain, belching, dysgeusia. The compliance rate of Group A and Group B was 93.7% and 95.9%, respectively (p=0.435).

**Table 2:** Gastrointestinal side effects of patients treated with probiotics (A) or placebo (B) during eradication therapy.

	Group A	Group B	*P* value
Total	30 (23.62%)	46 (37.70%)	0.016
Vomitting	3 (2.36%)	9 (7.38%)	0.065
Abdominal pain	21 (16.54%)	17 (13.93%)	0.568
Bloating	15 (11.81%)	21 (27.21%)	0.226
Nausea	17 (13.39%)	30 (24.59%)	0.024
Diarrhea	1 (0.79%)	1 (0.82%)	0.977
Belching	8 (6.30%)	12 (9.84%)	0.305
Dysgeusia	4 (3.15%)	4 (3.28%)	0.954

### Disturbance and restoration of gut microbiota after *H. pylori* eradication

**Figure 2:**
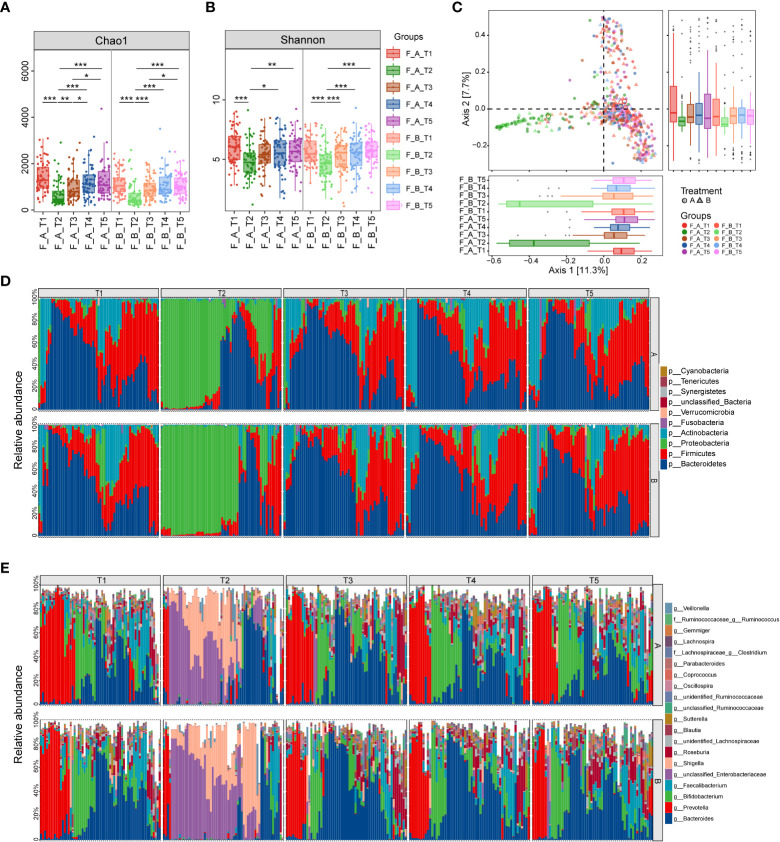
Comparison of diversity and composition of gut microbiota after eradication therapy. **(A, B)** Alpha diversity, representing the richness of microbiota, was significantly reduced immediately following eradication treatment (T2) and then gradually returned to baseline from T3 to T5. **(C)** Principal coordinate analysis (PCoA) based on Bray-Curtis distances revealed the overall microbiota composition differed between T2 and other time points. **(D)** Relative proportions of the top 10 bacterial phyla. **(E)** Relative proportions of the top 20 bacterial genera. A, probiotics group; B, placebo group; T1, pretreatment; T2, cessation of quadruple therapy; T3, 2 weeks after quadruple therapy; T4, 8 weeks after quadruple therapy; T5, 24 weeks after quadruple therapy; *, p<0.05; **, p<0.01, ***, p<0.001.

A total of 597 fecal samples were collected from 65 patients in Group A and 61 patients from Group B at different time points (**Figure 1**). The indices of alpha diversity, including Chao1 and Shannon, were remarkably reduced at week 2 (T2) compared to baseline (T1). Those indexes were quickly increased to the level of baseline at week 4 (T3), and maintained at the steady level until 1 year (T5) (**Figures 2A, B**). However, no significant difference of those indices was observed between Group A and Group B.

Principal coordinated analysis (PCoA) based on Bray-Curtis distance revealed that the overall microbial composition of T2 deviated from other time points (**Figure 2C**, PERMANOVA, p=0.001), although there was no statistical difference between Group A and Group B. Pairwise permanova analysis showed the difference of beta diversity between T3 and T1 was greater in Group B compared to Group A (PERMANOVA, A_T3 vs A_T1: p=0.049, B_T3 vs B_T1: p=0.037), and the distinction relative to baseline was disappeared at T4 in both groups.

**Figure 3:**
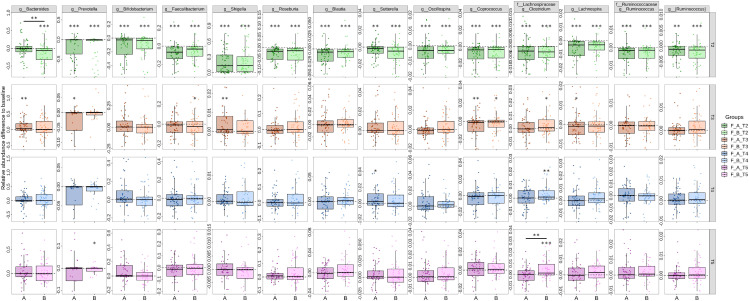
Dynamic alterations of genus-level gut microbiota after different periods of *H. pylori* eradication. 14 most abundant genera that differed between T1 and T2 were identified, and their alterations from T2 to T5 were calculated based on their abundance relative to T1. Value that lower than zero represents reduced relative abundance compared to baseline, whereas higher than zero represents increased relative abundance. *, p<0.05; **, p<0.01; ***, p<0.001; star without a line, group compared to T1; star with a line, comparison between **(A, B)**. **(A)** probiotics group; **(B)** placebo group; T1, pretreatment; T2, cessation of quadruple therapy; T3, 2 weeks after quadruple therapy; T4, 8 weeks after quadruple therapy; T5, 24 weeks after quadruple therapy.

To investigate the specific microbiota that contribute to the fluctuations, we assessed the relative abundance of taxa at different periods. At the phylum level, the gut microbiota is dominated by Bacteroidetes and Firmicutes before treatment, which account for up to 60% in most samples. Of note, the relative abundance of Proteobacteria was dramatically elevated as the dominant phylum following the cessation of eradication drugs, while Bacteroidetes, Firmicutes and Actinobacteria were significantly reduced (**Figure 2D**). The dominance of Proteobacteria was replaced with Bacteroidetes and Firmicutes from T3 to T5, indicating the compositional restoration to baseline. The proportion of patients dominated with Proteobacteria was higher in Group B compared to Group A. The most abundant genera were *Bacteroides, Prevotella, Bifidobacterium* and *Faecalibacterium* at baseline, which were substituted by *Shigella* and an unclassified genus in the family of *Enterobacteriaceae* at T2 (**Figure 2E**). The percentage of patients with prevailing *Shigella* and the unclassified genus of *Enterobacteriaceae* was larger in Group B compared to Group A. The relative abundance of *Bacteroides* was distinctly decreased in Group B relative to Group A, which indicate that probiotics could partially resist the destruction of eradication drugs on *Bacteroides* (**Figure 3**). The compositional disproportionation at T2, including the reduction of *Bifidobacterium*, *Faecalibacterium*, *Roseburia*, *Blautia*, etc, and over-representation of *Shigella* was gradually restored from T3 to T5 compared to baseline.

**Figure 4:**
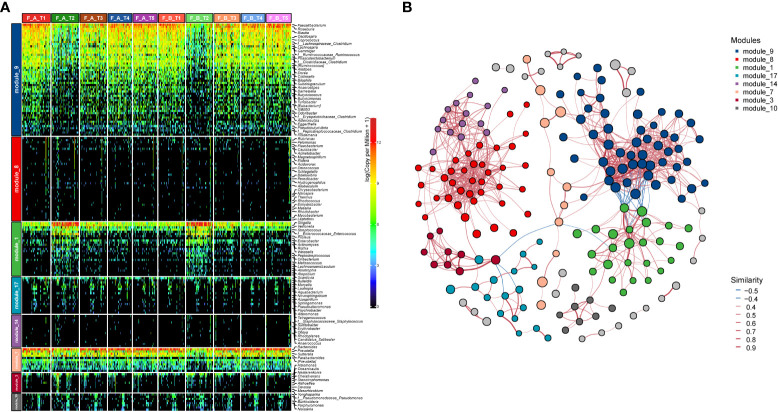
Co-occurrence network reveals the transformation of gut microbial pattern and their interconnection. **(A)** Heatmap shows the relative abundance of genera in each module. An obvious transformation of module_9 to module_1 was observed at T2, which was reverted from T3 to T5. **(B)** Significant co-occurrence and co-exclusion relationships among the modules. Each node represents a genus colored by module and its size is proportional to the relative abundance. Edge color indicates the positive (red) and negative (blue) association. The width of each line exhibits the correlation strength among genus.

We further performed co-occurrence network analysis to assess the impact of eradication treatment on gut microbiota interactions at different time points (**Figure 4**). The communities could be classified into eight modules with the prevalence of module_9, module_1 and module_7, while other modules distributed with individual variation. The community of module_9 was transformed to module_1 at T2 compared to T1, and subsequently restored from T3 to T5. The inter-correlations of taxa in module_9 was stronger than those in module_1. We also observed the co-occurring interactions between module_9 and module_7, while both of them, especially module_9, were co-excluded with module_1. Compared to baseline, the alterations of module_1 and module_7 at T2 were more evident in Group B compared to Group A.

### Probiotics mitigated alterations of gastric microbiota induced by *H. pylori* eradication

**Figure 5:**
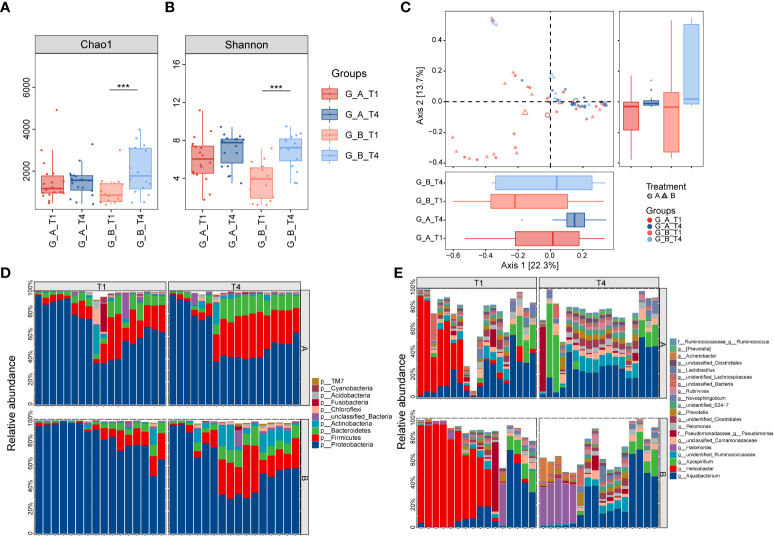
Longitudinal changes of gastric microbial diversity and composition after *H. pylori* eradication. **(A, B)** Box and whisker plots of the alpha diversity indices for richness and diversity of the bacterial communities on the ASV level in patients treated with either probiotics or placebo at T1 and T4, respectively. **(C)** Beta diversity analysis is represented by principal coordinate analysis based on Bray-Curtis distance. The red samples (T1) are deviated from the blue samples (T4). Relative abundance of the top 10 phyla **(D)** and the top 20 genera **(E)** in gastric mucosa. ***, p<0.001. A, probiotics group; B, placebo group; T1, pretreatment; T4, 8 weeks after quadruple therapy; T1, pretreatment; T4, 8 weeks after quadruple therapy.

To further explore the changes of gastric microbiota, a total of 68 paired gastric mucosa samples were collected from 34 patients, including 18 in Group A and 16 in Group B, before and 8 weeks after *H. pylori* treatment. An increased alpha diversity was observed after *H. pylori* eradication in Group B, measured by Chao1 and Shannon, while no significant change was observed in Group A (**Figures 5A, B**). PCoA based on Bray-Curtis distance revealed that the samples of T4 deviated in global microbiome structure from those of T1 (**Figure 5C**). PERMANOVA analysis demonstrated significant differences in gastric taxonomic composition between Group A and Group B at T4, while there was no obvious difference between these two groups at baseline.

**Figure 6:**
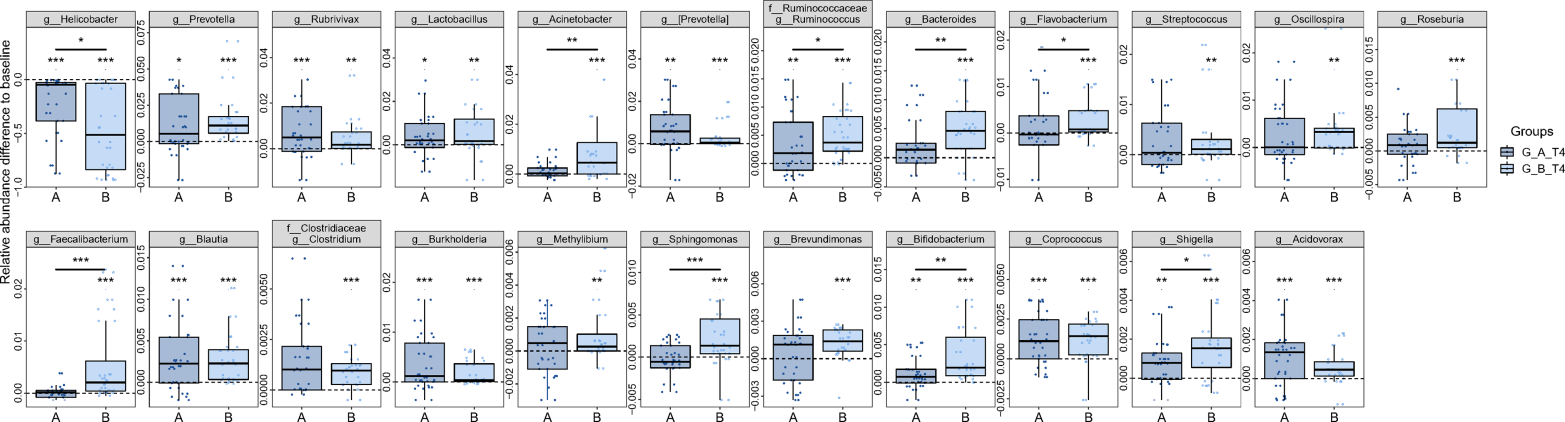
Alterations of distinct gastric genera after *H. pylori* eradication. The most abundant genera that differed between T1 and T4 were identified. The difference was illustrated based on the relative abundance of T4 compared to T1. Values above zero represent that the genera increased after therapy, whereas those below zero represent the decreased genera. *p<0.05; **p<0.01; ***p<0.001; star without a line, comparison between T1 and T4; star with a line, comparison between **(A, B)**; **(A)** probiotics group; **(B)** placebo group.

Different from gut microbiota, the gastric microbiota was predominated with Proteobacteria followed by Firmicutes as well as Bacteroidetes in patients with *H. pylori* infection (**Figure 5D**). With the reduction of Proteobacteria after eradication, the relative abundances of Firmicutes and Bacteroidetes were enhanced. At the genus level, *Helicobacter* was abundant at baseline albeit with inter-individual variation, and was approximately depleted after eradication, accompanied with the enrichment of *Lactobacillus*, *Prevotella*, *Bifidobacterium*, etc (**Figure 5E**). Notably, some taxa including *Faecalibacterium*, *Bacteroides*, *Streptococcus*, displayed differential abundance at T4 relative to T1 in Group B, while their abundances were not changed in Group A (**Figure 6**). Additionally, the amplitude of fluctuation was smaller in Group A compared to Group B, such as *Shigella*, *Ruminococcus*, *Bifidobacterium*. In order to investigate the changes of microbial dysbiosis after *H. pylori* eradication, Microbial Dysbiosis Index (MDI) were calculated using differential genera between T4 and T1. The shift of MDI from T1 to T4 was greater in Group B compared to Group A (**Supplement Figure 1A**). The delta MDI of Group B was farther to zero than Group A, indicating that probiotics play a role in attenuating the turbulence of gastric microbiota after *H. pylori* eradication (**Supplement Figure 1B**). The co-occurrence network analysis showed that module_2, module_5 and module_3 were the predominant communities in the stomach, albeit with distinct intraindividual variation (**Supplement Figure 2**). Negative correlation was observed between module_2 and module_5, while module_2 was co-occurred with module_4 and module_3, although relatively weak. The changes of module_2 and module_5 between T4 and T1 were remarkable in Group B compared to Group A.

### Changes of saliva microbiota after *H. pylori* eradication

**Figure 7:**
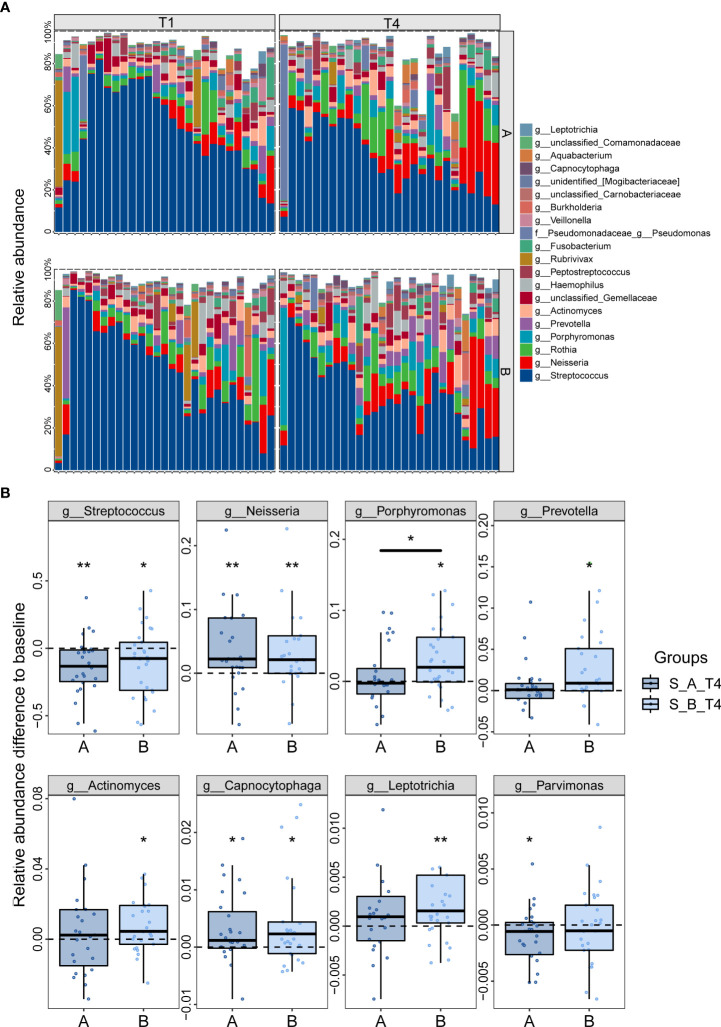
Effect of probiotics on saliva microbiota in patients following *H. pylori* eradication. **(A)** Compositional changes of the top 20 most abundant saliva genera. **(B)** Alterations of differential genera after *H. pylori* eradication. Values above zero represented the increased relative abundance at T4 relative to baseline, while those below zero represented decreased abundance. *p<0.05; **p<0.01; star without a line, comparison between T1 and T4; star with a line, comparison between **(A, B)**; **(A)** probiotics group; **(B)** placebo group.

Since the oral cavity is the entry port and the first component of the gastrointestinal system, the colonization of *H. pylori* in the stomach has been supposed to derive from a potential oral-oral transmission route. Thus, we explored the oral prevalence of *H. pylori* and the alterations of oral microbiota after eradication. 112 matched-saliva samples were collected from 56 patients (n=27 for Group A, n=29 for Group B) before and after 8 weeks of eradication therapy. The alpha diversity indexes showed no significant difference among the four groups (**Supplement Figure 3A**). Significant shift of the overall microbial structure, illustrated by PCoA analysis, was observed in Group B rather than Group A (**Supplement Figure 3B**). The oral microbiota is mainly composed of *Streptococcus*, *Neisseria*, *Rothia*, *Porphyromonas*, which account for more than 50% of the taxa detected (**Figure 7A**). The relative abundance of *H. pylori* in the saliva was extremely low and showed no correlation with the gastric *H. pylori* (**Supplement Figure 4**). The eradication of gastric *H. pylori* did not influence the amount of *H. pylori* in the saliva. While *Streptococcus* was significantly decreased at T4 compared to baseline, the amount of *Neisseria* was enriched (**Figure 7B**). Moreover, some pathogenic taxa including *Porphyromonas*, *Leptotrichia*, *Actinomyces*, *Prevotella* elevated significantly in Group B after eradication, yet their abundance remained at pretreatment level in Group A.

## Discussion

**Figure 8:**
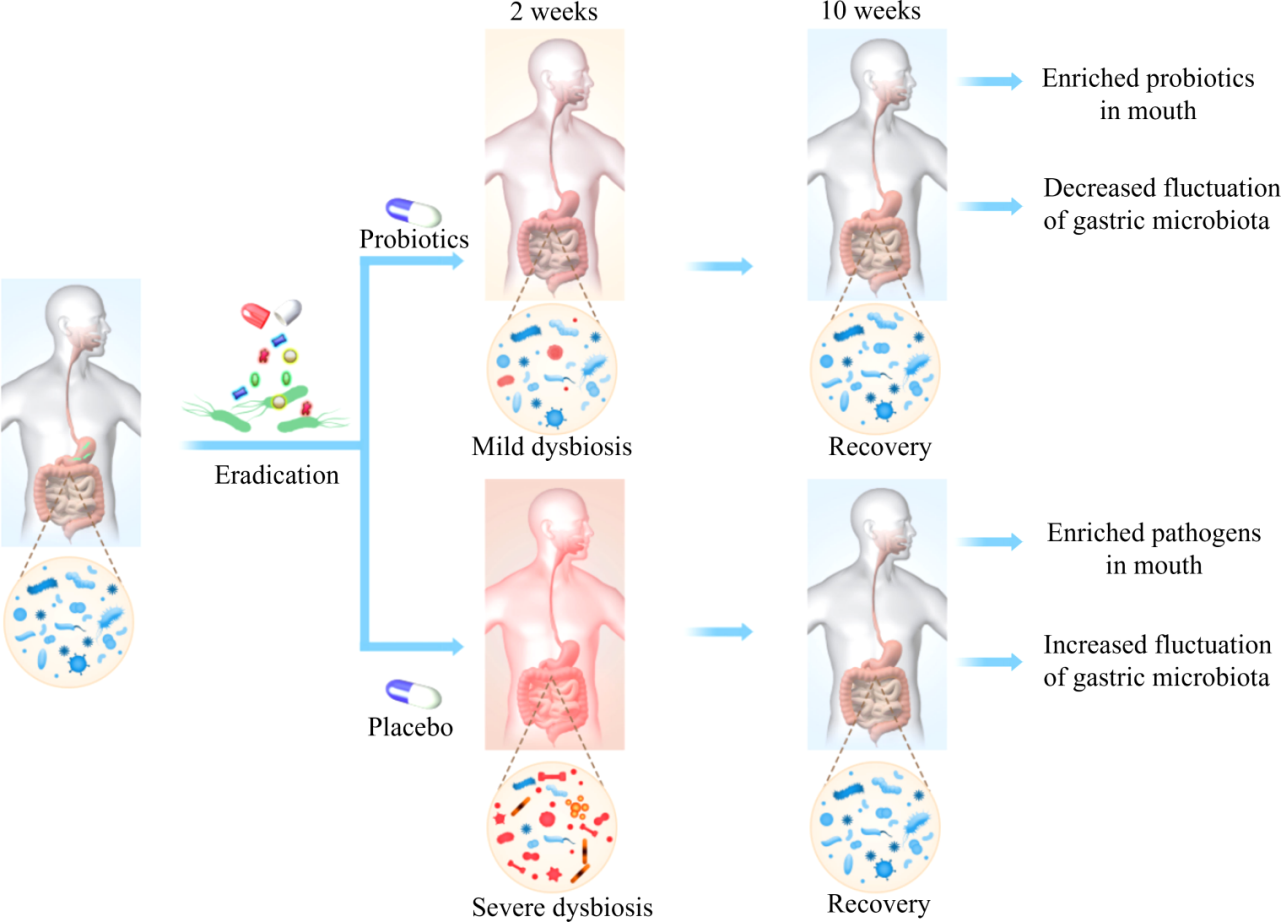
There were dramatical changes in gut microbiota immediately after *H. pylori* eradication, which were swiftly restored after 8 weeks. These perturbations were partially counteracted by probiotics. Meanwhile, alterations of oral and gastric microbiota were also observed. Probiotics mitigated the fluctuations of gastric microbiota as well as promoted the enrichment of beneficial bacteria in the mouth.

To our knowledge, this is the first multi-center, randomized, double-blinded, placebo-controlled trial to show the effect of probiotics (Bifidobacterium Tetravaccine Tablets) combined with quadruple Bismuth-containing regimen on *H. pylori* eradication rate, gastrointestinal adverse events (GAE), as well as microbiota along gastrointestinal tract. The probiotics adjuvant therapy reduced the incidence of GAE, while showed no effect on eradication rate. The composition of microbial community across saliva, stomach and gut changed significantly after eradication, with the gut microbiota swiftly restored to baseline as soon as two weeks after cessation of antibiotics. Interestingly, the disturbance of microbiota induced by eradication drugs could be partially alleviated by probiotics treatment (**Figure 8**).

The infection of *H. pylori* is associated with a variety of diseases, including gastritis, peptic ulcers and gastric cancer. Therefore, a subsequent of global consensus has recommended eradication therapy, unless there are competing considerations (1, 20, 21). With the prevalence of antibiotic resistance worldwide, the first-line regimen transformed from triple to quadruple therapy and the duration was prolonged from 7 days to 14 days, causing increased side effects and poor compliance. Recently, a large number of studies explored the efficacy of adjuvant probiotics therapy for patients with *H. pylori* infection, yet the clinical outcomes were controversial (3). While some studies reported improved eradication rate in patients given probiotics, others showed negative results (10, 22). We observed no difference between probiotics and placebo group in eradication rate, both of which achieved up to 90% as revealed by PP analysis. Additionally, we found that probiotics combination mitigated gastrointestinal adverse events induced by eradication therapy, which is in agreement with other studies (10, 12).

Accumulating evidence suggest that *H. pylori* therapy can lead to short-term impairment of gut microbiota, including decreased diversity and increased abundance of Proteobacteria (7). In line with previous reports, we observed a significant shift of gut microbiota immediately following treatment, and then began to recover after 2 weeks, indicating the resilience of the gut ecosystem. To further counteract the perturbations of gut microbiota caused by antibiotics involved in the eradication regimen, several studies investigated the effects of probiotics, such as *Enterococcus faecium*, *Bacillus subtilis*, *Lactobacillus reuteri (* 12, 23). The probiotic compound consumed in this study contains four live strains, which was different from previous studies. Despite the overall structure of gut microbiota was not different between probiotic and placebo group, the genus *Bacteroides* was more abundant in patients treated with probiotics compared to placebo. As commensals and mutualists, *Bacteroides* establish stable, long-term associations with human hosts and confer numerous health benefits, including anti-inflammation, metabolic regulation as well as cancer prevention (24). Nevertheless, the expansion of pathogenic Proteobacteria after cessation of antibiotics was not counteracted by probiotics. The co-occurrence network analysis also showed the wane of commensal bacteria and the wax of pathogens such as *Shigella* after eradication no matter with or without probiotics. We speculate that the activity of probiotics was impaired by antibiotics as the probiotic strains were not detected in fecal samples. Thus, it is necessary to improve the type and dose of probiotic strains in the future in addition to the staggered dosing. The commensal bacteria including *Faecalibacterium, Roseburia, Blautia*, which were prevalent at baseline, are potential candidates for the development of novel probiotic.

Recent advances in sequencing technology enabled to identify a complex microbiota within the human stomach in addition to *H. pylori* that may be potentially associated with various disease states (25). Most reports show that *H. pylori*-infected patients exhibited unbalanced gastric microbiota compared to the negative individuals, which was reverted after eradication therapy (19). Our observation agreed with previous reports that show the depletion of one genus *Helicobacter* and the over-representation of other taxa such as *Streptococcus*, *Prevotella*, *Bacteroides*, et al. after treatment. Of interest, we found that the fluctuation of gastric microbiome was milder in probiotics group compared to those received placebo, indicating the ability of probiotics on keeping equilibrium of the ecosystem during the exposure of antibiotics. Consistently, several studies have demonstrated the modulation of probiotics on gastric microbiota (14, 26). We speculate that the reduced incidence of upper gastrointestinal side effects such as nausea and vomiting might be associated with the homeostasis of gastric microbiota in patients with probiotics consumption.

A potential oral-oral transmission route of *H. pylori* raises the question concerning whether it colonizes in the oral cavity. Our study showed very low levels of *H. pylori* in the saliva from patients with confirmed gastric *H. pylori* infection, and there was no significant change after eradication therapy. This observation has been supported by a previous study (27). Additionally, we found that *H. pylori* eradication brought about reduction of *Streptococcus* together with noteworthy elevation of *Neisseria*. Furthermore, probiotics influenced the oral microbiota by suppressing the expansion of some taxa, including *Porphyromonas*, *Leptotrichia*, and *Actinomyces*. The nitrate-reducing bacteria *Neisseria* are consistently found at higher levels in individuals with healthy oral status and linked with better cardiometabolic health (28). On the other hand, *Streptococcus*, *Porphyromonas*, *Actinomyces* were reported to be associated with dental caries and gingivitis (29).

Although our investigations attempt to provide a comprehensive insight into the gastrointestinal microbiome alterations after *H. pylori* eradication as well as the impact of probiotics on microbial configuration, there are several limitations to be addressed. First, we did not collect samples from *H. pylori* negative individuals. It is difficult to determine whether the eradication therapy restored the dysbiosis of gastrointestinal microbiota induced by *H. pylori* infection towards healthy status. Second, we were not able to elucidate the long-term effect of *H. pylori* eradication on gastric and oral microbiota, as the samples were collected after merely 2 months. Third, the samples collected at different sites were not identical to the number of patients enrolled due to the low compliance.

## Conclusion

Our data reported the reconstitution of gastric and oral microbiota after *H. pylori* depletion, accompanied with transient perturbations of gut microbiota that generally returned to baseline by 2 weeks. The alterations of microbiota were not only observed in the gut but also saliva and stomach after probiotics treatment with different variations. These results highlight a need of developing high efficacy probiotics that target multiple parts of microbiota to improve resilience of the microbial ecosystem during disruptions and to restore its equilibrium afterwards.

## Data availability statement

The raw Illumina sequencing data have been deposited in the NCBI Sequence Read Archive (SRA, http://www.ncbi.nlm.nih.gov/sra) under accession number PRJNA839582 for saliva samples, PRJNA801428 for gastric mucosa samples, PRJNA801350 for fecal samples.

## Ethics statement

This study was approved by the Ethics Committee of the First Affiliated Hospital of Nanchang University (2019-072) and registered at ClinicalTrials.gov (NCT04034641). The patients/participants provided their written informed consent to participate in this study. Written informed consent was obtained from the individual(s) for the publication of any potentially identifiable images or data included in this article.

## Author contributions

All authors have read and approved the final manuscript NL, BL, ZZ designed and supervised the project CH performed bioinformatics analysis and drafted the manuscript YX and YZ collected samples, recorded clinical data and analyzed the clinical parameters KZ, LH, YY, QG, XS, ZX performed clinical diagnosis and enrolled eligible patients. All authors contributed to the article and approved the submitted version.

## Conflict of interest

The authors declare that the research was conducted in the absence of any commercial or financial relationships that could be construed as a potential conflict of interest.

## Publisher’s note

All claims expressed in this article are solely those of the authors and do not necessarily represent those of their affiliated organizations, or those of the publisher, the editors and the reviewers. Any product that may be evaluated in this article, or claim that may be made by its manufacturer, is not guaranteed or endorsed by the publisher.
