# Supplementary material for: Probiotics modulate gastrointestinal microbiota after Helicobacter pylori eradication: A multicenter randomized double-blind placebo-controlled trial
Source: Front Immunol. 2022 Nov 8;13:1033063. doi: 10.3389/fimmu.2022.1033063 (PMC9679295; doi:10.3389/fimmu.2022.1033063)
Supplement: Supplementary file 1 [file DataSheet_1.docx]

Supplement materials

Methods

Randomization and masking

Participants were randomly assigned in a 1:1 ratio to receive the probiotic product or placebo. The randomization into one of the two study groups was done in blocks of 4 using a computerized random number generator. The sequence was generated by an independent statistician who did not involve in the study. The study products were labelled according to the numbers in the randomization list that was stored in sealed envelopes and stored at the First Affiliated Hospital of Nanchang University. Both probiotic and placebo were identical in appearance, taste and texture. The database was locked after the completion of clinical observation and the assignment was revealed to the statistician responsible for data analysis.

Bacterial DNA extraction and 16S rRNA gene sequencing

Total genomic DNA was extracted using the OMEGA Soil DNA Kit (M5635-02) (Omega Bio-Tek, USA) according to manufacturer’s protocols, and then stored at -20℃. The quality and quantity of the DNA samples were measured using agarose gel electrophoresis and NanoDrop NC2000 spectrophotometer (Thermo Fisher Scientific, USA), respectively.

The V3-V4 hypervariable regions of the 16S rRNA gene were amplified by polymerase chain reaction (PCR) using the following primers: 338F (5’-ACTCCTACGGGAGGCAGCA-3’) AND 806R (5’-GGACTACHVGGGTWTCTAAT-3’). Sample specific 7-bp barcodes were incorporated into the primers for multiplex sequencing. The reaction volume (25μl) is comprised of 5x reaction buffer (5μl), 5U/μl Fast pfu DNA Polymerase (0.25μl), 2.5mM dNTPs (2μl), 10μM each primer (1μl), DNA Template (1μl), and ddH_2_O (14.75μl). Cycling process consisted of initial denaturation at 98℃ for 5 min, followed by 25 cycles of denaturation at 98℃ for 30 s, annealing at 53℃ for 30 s, extension at 72℃ for 45 s, with a final extension at 72℃ for 5 min. PCR amplicons were purified with Vazyme VAHTSTM DNA Clean Beads (Vazyme, China), and quantified using the Quant-iT PicoGreen dsDNA Assay Kit (Invitrogen, USA). Quantified amplicons were pooled in equal amounts and pair-end 2x250 bp sequencing was performed using the Illumina NovaSeq platform with NovaSeq 6000 SP Reagent Kit (500 cycles) (Shanghai Personal Biotechnology Co., Ltd, China).

Bioinformatic analysis

Microbiome bioinformatic analysis was performed by QIIME2 2019.4 with slight modification according to the official tutorials[1]. Briefly, raw sequence data were demultiplexed using the demux plugin following by primers cutting with cutadapt plugin[2]. Sequences were then quality filtered, denoised, merged and chimera removed using DADA2[3]. Non-singleton amplicon sequences variants (ASVs) were aligned with mafft and used to construct a phylogeny with fasttree2[4, 5]. Taxonomy was assigned to ASVs using the classify-sklearn naïve Bayes taxonomy classifier in feature-classifier against the Greengenes 13.8[6, 7].

Statistical analysis

Statistical analysis was mainly performed using QIIME2 and R packages (v3.6.0). Alpha diversity of microbial community was estimated using Chao1 and Shannon index and beta diversity was calculated by principal coordinate analysis based on Bray-Curtis distance. Wilcoxon Signed Rank-Sum test was performed to compare the difference of alpha diversity indices, and significant difference of beta diversity was evaluated by PERMANOVA. Only taxa with frequencies of sample occurrence not less than 40%, were chosen to perform differential analysis. Genera with significantly changed abundance in T4 were combined into Microbial Dysbiosis Index (MDI) for using follow formula: MDI=log(total abundance of genera decreased after *H. pylori* eradication/total abundance of genera increased after *H. pylori* eradication)[8]. The difference between the MDI of the timepoint of T4 and the baseline T1 were defined as delta-MDI, which was calculated using the difference value of MDI between paired samples. Co-occurrence network analysis was performed using the function *corrr.test* in R package psych with the spearman’s rank correlation analysis setting as the correlation calculation method. The “fdr” adjustment was used for multiple test p value corrections. The cutoffs of the adjusted p values and the correlation coefficients were set as 0.01 and 0.4. Based on the correlation coefficients, we constructed co-occurrence network with nodes representing ASVs and edges representing correlations between these ASVs. Network was visualized using R package igraph and ggraph. Pearson correlation analysis was used to analyze the relationship between gastric and saliva *H. pylori* abundance.

Referrence

1 Bolyen E.; Rideout J.R.; Dillon M.R.; et al. Reproducible, interactive, scalable and extensible microbiome data science using QIIME 2. *Nat Biotechnol* **2019**;37:852-7.

2 Kechin A.; Boyarskikh U.; Kel A.; et al. cutPrimers: A New Tool for Accurate Cutting of Primers from Reads of Targeted Next Generation Sequencing. *J Comput Biol* **2017**;24:1138-43.

3 Callahan B.J.; McMurdie P.J.; Rosen M.J.; et al. DADA2: High-resolution sample inference from Illumina amplicon data. *Nat Methods* **2016**;13:581-3.

4 Katoh K.; Misawa K.; Kuma K.; et al. MAFFT: a novel method for rapid multiple sequence alignment based on fast Fourier transform. *Nucleic Acids Res* **2002**;30:3059-66.

5 Price M.N.; Dehal P.S.; Arkin A.P. FastTree: computing large minimum evolution trees with profiles instead of a distance matrix. *Mol Biol Evol* **2009**;26:1641-50.

6 Bokulich N.A.; Kaehler B.D.; Rideout J.R.; et al. Optimizing taxonomic classification of marker-gene amplicon sequences with QIIME 2's q2-feature-classifier plugin. *Microbiome* **2018**;6:90.

7 Koljalg U.; Nilsson R.H.; Abarenkov K.; et al. Towards a unified paradigm for sequence-based identification of fungi. *Mol Ecol* **2013**;22:5271-7.

8 Guo Y.; Zhang Y.; Gerhard M.; et al. Effect of Helicobacter pylori on gastrointestinal microbiota: a population-based study in Linqu, a high-risk area of gastric cancer. *Gut* **2020**;69:1598-607.
