# Supplementary material for: Probiotics modulate gastrointestinal microbiota after Helicobacter pylori eradication: A multicenter randomized double-blind placebo-controlled trial
Source: Front Immunol. 2022 Nov 8;13:1033063. doi: 10.3389/fimmu.2022.1033063 (PMC9679295; doi:10.3389/fimmu.2022.1033063)
Supplement: Supplementary file 2 [file DataSheet_2.docx]

Supplement figure legends

Supplement figure 1 Probiotics mitigated gastric microbial dysbiosis index (MDI) induced by *H. pylori* eradication. MDI was calculated to reflect the fluctuation of gastric microbiota after *H. pylori* eradication (A) and the variation relative to baseline was greater in Group B compared to Group A (B). **, p<0.01; ***, p<0.001. A, probiotics group; B, placebo group; T1, pretreatment; T4, 8 weeks after quadruple therapy.

Supplement figure 2 Co-occurrence networks of gastric microbiota after *H. pylori* eradication in patients given probiotics or placebo. (A) Heatmap showed the relative abundance of genera in each core module. The differences between T1 and T4 were more remarkable in Group B compared to Group A. (B) Co-occurrence and co-excludence relationships among each module. Node sizes were proportional to relative abundance of each genus, and different colors represented the modules within networks. The width of lines represented the correlation strength, and colors represented positive (red) or negative (blue) correlation. A, probiotics group; B, placebo group; T1, pretreatment; T4, 8 weeks after quadruple therapy.

Supplement figure 3 The diversity of saliva microbiota in patients after *H. pylori* eradication. (A) Alpha diversity indexes, including Chao1 and Shannon, were calculated before and after *H. pylori* eradication. (B) PCoA plot of the saliva microbiota based on Bray-Curtis distance. A, probiotics group; B, placebo group; T1, pretreatment; T4, 8 weeks after quadruple therapy.

Supplement figure 4 No significant correlation was observed between gastric and saliva *H. pylori* as revealed by Pearson analysis.
