# Supplementary figures and images for: Probiotics modulate gastrointestinal microbiota after Helicobacter pylori eradication: A multicenter randomized double-blind placebo-controlled trial
Source: Front Immunol. 2022 Nov 8;13:1033063. doi: 10.3389/fimmu.2022.1033063 (PMC9679295; doi:10.3389/fimmu.2022.1033063)

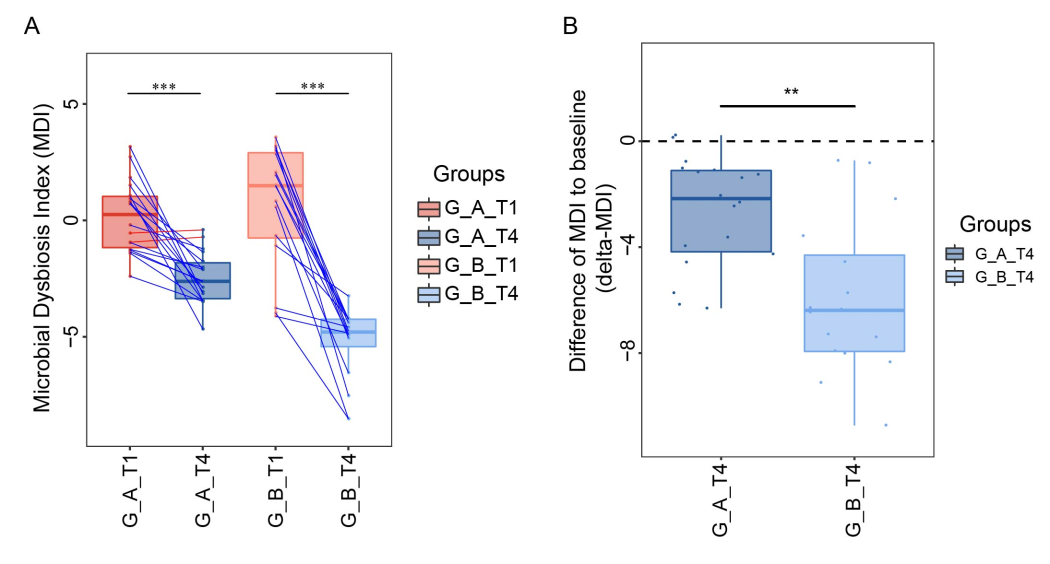

Supplement: Supplementary file 3 [file Image_1.tiff]

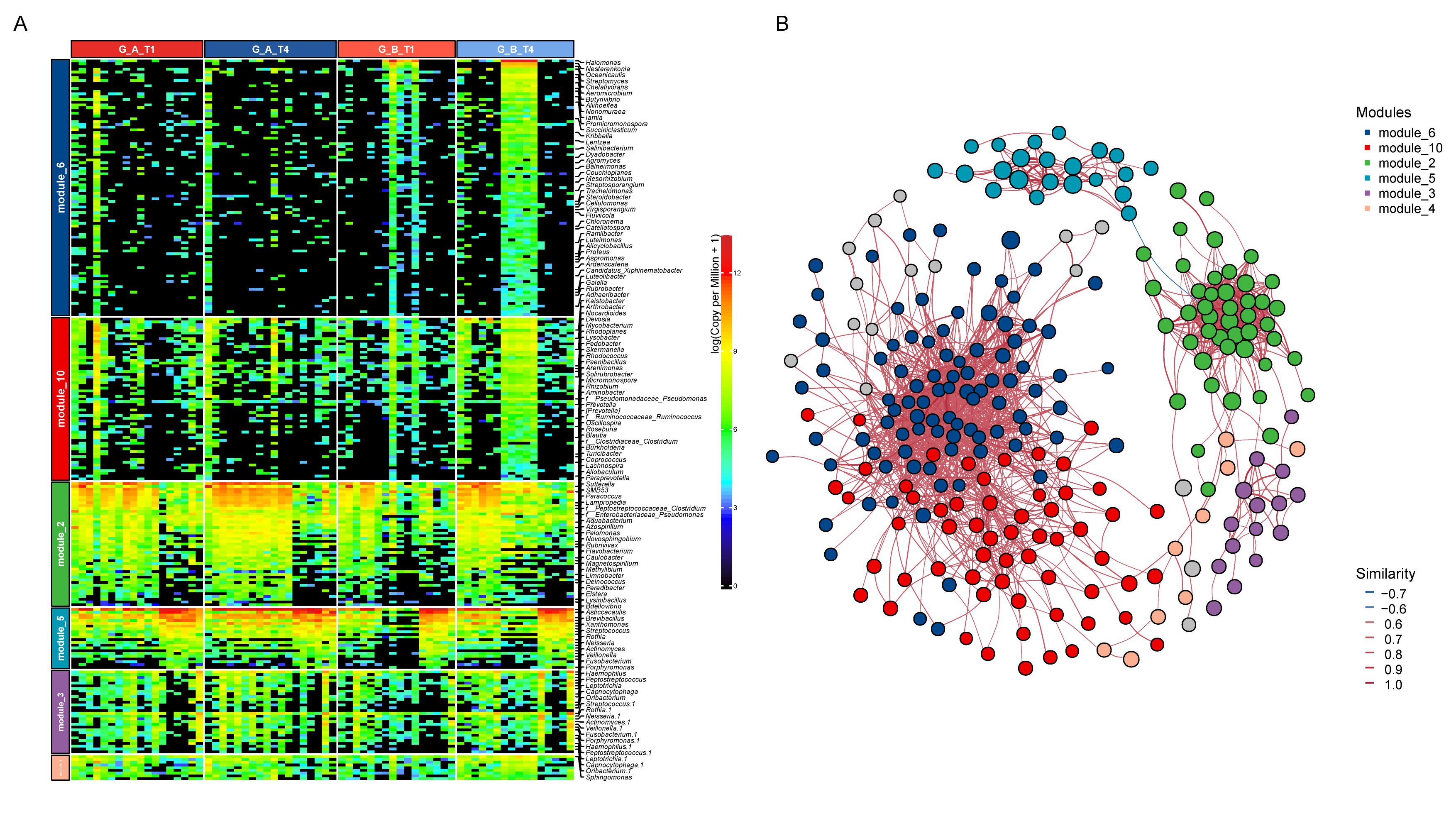

Supplement: Supplementary file 4 [file Image_2.tif]

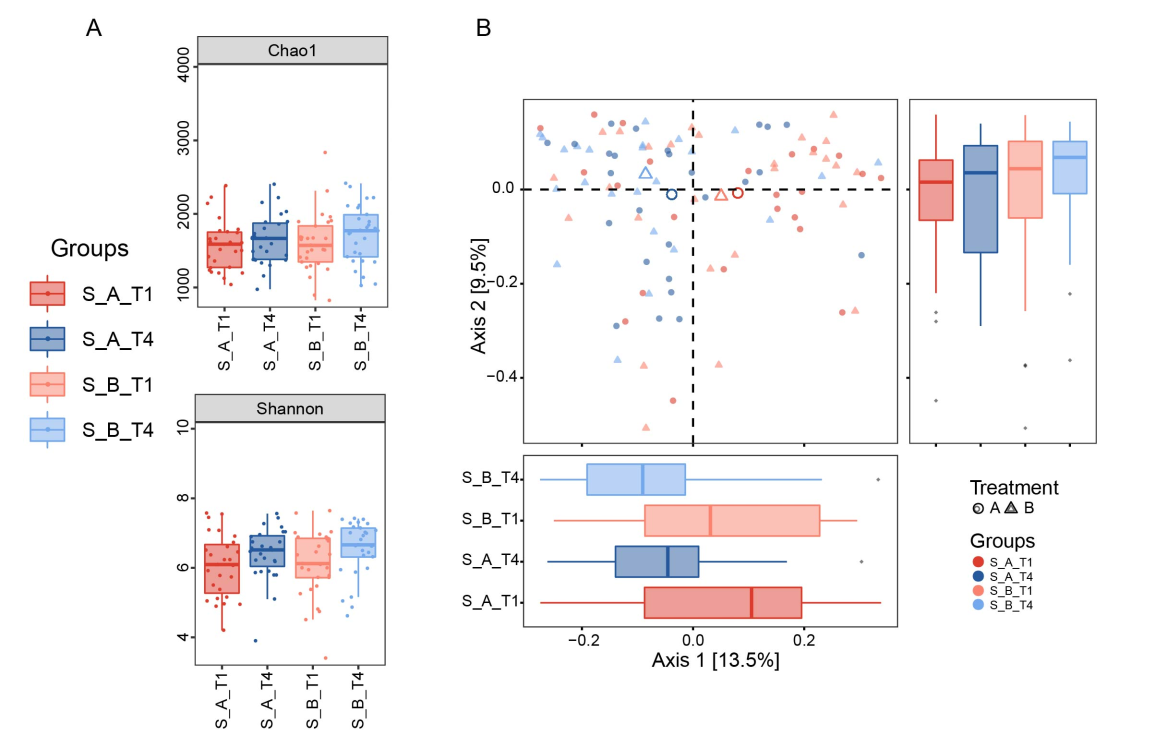

Supplement: Supplementary file 5 [file Image_3.tiff]

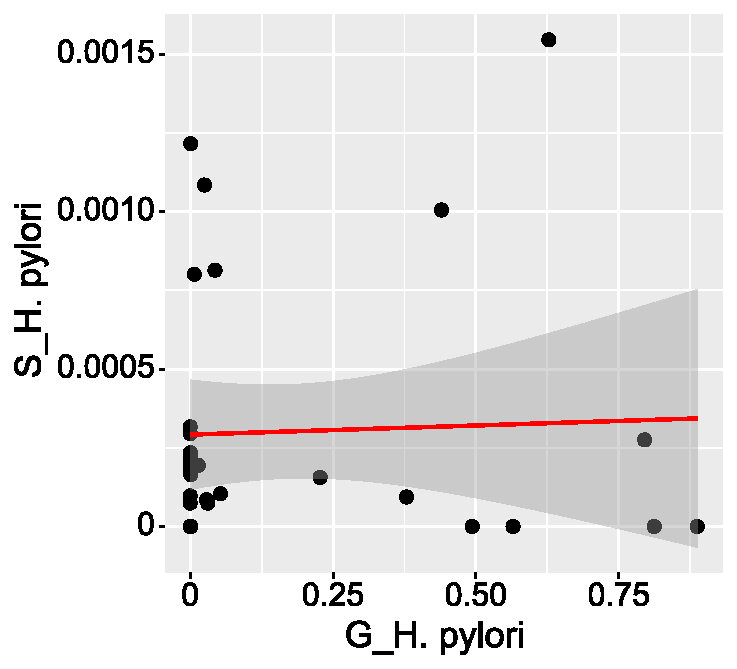

Supplement: Supplementary file 6 [file Image_4.tiff]

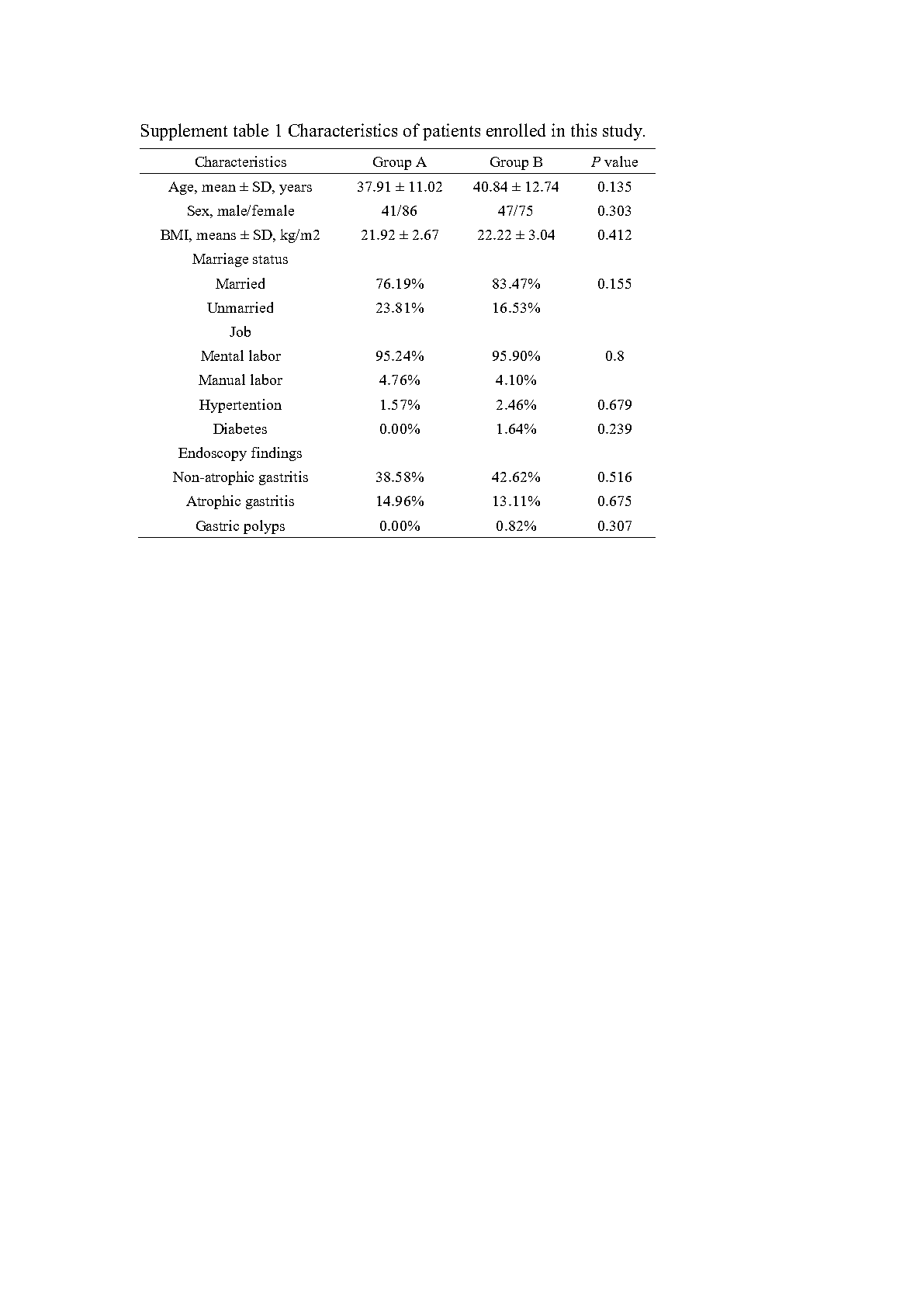

Supplement: Supplementary file 7 [file Table_1.docx]
